# The association between waiting time and multidisciplinary pain treatment outcomes in patients with rheumatic conditions

**DOI:** 10.1186/s41927-020-00157-0

**Published:** 2020-10-23

**Authors:** Simon Deslauriers, Jean-Sébastien Roy, Sasha Bernatsky, Debbie E. Feldman, Anne Marie Pinard, François Desmeules, Mary-Ann Fitzcharles, Kadija Perreault

**Affiliations:** 1 Center for Interdisciplinary Research in Rehabilitation and Social Integration (CIRRIS), 525, boulevard W.-Hamel, Quebec, QC G1M 2S8 Canada; 2 Faculty of medicine, Université Laval, CHUL, 2705, boulevard Laurier, #3412, Quebec, QC G1V 4G2 Canada; 3 McGill University Health Centre (MUHC), 1650 Cedar Ave, Montreal, QC H3G 1A4 Canada; 4 McGill University, Montréal, Canada; 5 Research Institute of the McGill University Health Centre (RI-MUHC), Montréal, Canada; 6 Faculty of medicine, Université de Montréal, Montreal, Canada; 7 Centre for Interdisciplinary Research in Rehabilitation of Greater Montreal (CRIR), CRIR, 6363, chemin Hudson (Pavillon Lindsay) bureau 061, Montréal, QC H3S 1M9 Canada; 8 Public Health Research Institute of Université de Montréal, Montréal, Canada; 9 Centre hospitalier universitaire (CHU) de Québec, Québec, Canada; 10 Maisonneuve-Rosemont Hospital (CRHMR) Research Center, CRHMR, 5415 Assomption boulevard, Montreal, QC H1T 2M4 Canada

**Keywords:** Waiting time, Clinical outcomes, Rheumatic conditions, Chronic pain, Multidisciplinary pain treatment facilities

## Abstract

**Background:**

Access to multidisciplinary pain treatment facilities (MPTF) is limited by extensive waiting time in many countries. However, there is a lack of knowledge about the impact of waiting time on clinical outcomes, particularly for patients with rheumatic conditions. This study examined the association between waiting time for MPTF and clinical outcomes in patients with rheumatic conditions.

**Methods:**

Data were extracted from the Quebec Pain Registry, a large database of patients who received services in MPTF. The associations between waiting time (classified as < 2 months, 2–6 months and >  6 months) and change in pain interference, pain intensity and health-related quality of life, from the initial visit at the MPTF to the 6-month follow-up, were tested using generalized estimating equations.

**Results:**

A total of 3230 patients with rheumatic conditions (mean age: 55.8 ± 14.0 years; 66% were women) were included in the analysis. Small significant differences in improvement between waiting time groups were revealed, with patients waiting less than 2 months having a larger improvement in all clinical outcomes compared to patients who waited 2–6 months or over 6 months before their initial visit (adjusted time X group effect *p* ≤ 0.001). Only patients waiting less than 2 months reached a clinically important improvement in pain interference (1.12/10), pain intensity (1.3/10) and physical and mental quality of life (3.9 and 3.7/100).

**Conclusions:**

Longer delays experienced by patients before receiving services in MPTF were associated with statistically significant smaller improvements in pain interference, pain intensity and health-related quality of life; these differences were, however, not clinically significant. Based on these results, we advise that strategies are developed not only to reduce waiting times and mitigate their impacts on patients with rheumatic conditions, but also to improve treatment effectiveness in MPTF.

## Background

Rheumatic conditions, which include autoimmune (e.g., rheumatoid arthritis), inflammatory (e.g., gout), degenerative conditions (e.g., osteoarthritis) and widespread body pain (e.g., fibromyalgia) [1], affect approximately one fifth of North American and European populations [2, 3]. In most rheumatic conditions, pain is a chief complaint and represents a major source of disability and worsening of health status [4, 5]. Effective pain treatment is thus a key component of the management of these conditions.

For many patients with rheumatic conditions, the biopsychosocial and chronic nature of their pain condition may require a multidisciplinary pain management approach combining pharmacological (e.g., non-steroidal anti-inflammatory drugs, acetaminophen, disease-modifying antirheumatic drugs) and non-pharmacological interventions (e.g., exercise, cognitive behavioral therapy, mindfulness-based stress reduction and other physical therapies) [6]. Evidence supports the benefits of these approaches for persons with rheumatic conditions to improve their pain, health-related quality of life, self-efficacy and overall physical condition [7, 8].

Multidisciplinary pain management programs can be delivered in multidisciplinary pain treatment facilities (MPTF), where integrated and patient-centered care is provided by different health professionals in order to improve patients’ pain, disability, quality of life and empowerment [9]. However, access to MPTF is limited by extensive waiting lists in many countries [10–12]. A recent study found an average waiting time of 8 months in MPTF in Quebec (Canada) for patients with rheumatic conditions for the period of 2008 to 2014 [13]. Therefore, a large number of patients with rheumatic conditions have to wait several months or even years before accessing services in MPTF. A systematic review found a deterioration in the health-related quality of life and psychological distress in patients with different chronic pain conditions after waiting 6 months for chronic pain treatment [14]. Yet, there remains a lack of knowledge about waiting time to access MPTF service and its impact on clinical outcomes, particularly for patients with rheumatic conditions. The primary objective of this study was to examine, in patients with rheumatic conditions, the association between waiting time to access MPTF and multidisciplinary pain treatment outcomes. A secondary objective was to determine if the association between waiting time and clinical outcomes was different for specific rheumatic conditions, including osteoarthritis and fibromyalgia.

## Methods

### Data source

Data used in this retrospective study were extracted from the Quebec Pain Registry, a large database of patients with chronic non-cancer pain who received services within five MPTF in the province of Québec, Canada. Consecutive patients aged 18 years and over who consented to participate were enrolled in the registry. Patients unable to understand written and spoken French or English or patients unable to participate due to severe physical or cognitive impairments were excluded. The registry includes data collected between 2008 and 2014 using self-administered questionnaires, structured interviews with a research nurse who had received a training specific to the registry, and a physician assessment. The self-administered questionnaires and the nurse interview were completed just prior to the initial visit at the MPTF and a follow-up was conducted 6 months after the initial visit. Patients enrolled in the registry usually received a combination of medical (e.g., nerve block), psychological (e.g., psychotherapy), physical (e.g., physiotherapy) or self-management interventions that were personalized to their needs [15, 16]. Detailed recruitment and data collection methods used in the Quebec Pain Registry are described elsewhere [16].

### Study population

Patients from the Quebec Pain Registry were included in this study if they 1) had received a diagnosis of a rheumatic condition by the referring or the MPTF’s physician (formal rheumatic diagnosis made by the physician), 2) self-reported having a rheumatic condition as a comorbidity or 3) reported a rheumatic condition was the cause of their pain (e.g., pain onset due to ankylosing spondylitis). This combination of physician-diagnosed and self-reported rheumatic conditions allowed to select a relatively exhaustive, although less specific, sample of patients with rheumatic conditions. In this study, ‘rheumatic conditions’ were defined using the National Arthritis Data Workgroup (NADW) case definition recommended for health services research [17] that provides a list of arthritis diagnostic codes from the 9th edition of the International Classification of Diseases (ICD-9-CM) system. While the NADW definition has not been updated with more recent ICD versions (ICD-10 or ICD-11), it remains frequently used and demonstrates high sensitivity [18], thus optimizing case detection [19]. The U.S. Centers for Disease Control and Prevention divide the NADW diagnostic codes further into the following categories: rheumatoid arthritis; fibromyalgia, myalgia and myositis; osteoarthritis and allied disorders; spondylarthropathy; gout and other crystal arthropathies; diffuse connective tissue disease; carpal tunnel syndrome; soft tissue disorders, excluding back; joint pain, effusion and other unspecified joint disorders; other specified rheumatic conditions [20].

### Variables

Waiting time (independent variable) was defined as the period between receipt of the referral and the first patient’s visit at the MPTF, which were all recorded by a research nurse in the baseline questionnaire. Considering the usual non-normal distribution of waiting time data (positively skewed with multiple outliers), the sample of patients with rheumatic conditions was divided into the following waiting time categories: less than 2 months, 2 to 6 months and more than 6 months. The 2 months cutoff was based on the International Association for the Study of Pain’s benchmark for chronic pain treatment [21], while the 6-month threshold was based on the results of Lynch et al.’s systematic review, which found a deterioration in patients’ quality of life after a 6-month waiting time for chronic pain treatment [22]. In addition, the use of a categorical variable facilitates the interpretation of the results.

The outcomes (dependent variables) were changes in pain interference, pain intensity and health-related quality of life between the initial MPTF visit and the 6-month follow-up. Measures used in this study follow the recommendations of the Initiative on Methods, Measurement, and Pain Assessment in Clinical Trials [23]. The ten items of the Brief Pain Inventory (BPI) included in the Quebec Pain Registry measure interference of the patients’ pain on various domains of their life, each rated on a 0 (pain does not interfere) to 10 (fully interferes) numeric scale [24]. A global 0–10 score of pain interference is derived by averaging the scores of the ten items. In patients with arthritis or chronic pain, the BPI has shown high internal consistency, good construct validity and adequate sensitivity to change [24, 25]. The minimal clinically important difference (MCID) in BPI is approximately 1 point [26].

The average pain intensity in the past week was obtained from the numeric pain rating scale (NPRS), a reliable and valid assessment tool for populations with rheumatic conditions [27]. The NPRS is an 11-item scale with 0 described as no pain at all and 10 the worst possible pain. In patients with chronic musculoskeletal pain, the MCID in the NPRS is approximately 1 point or a 15% change [28].

Health-related quality of life was measured by the self-administered 12-item Short-Form Health Survey version 2 (SF-12v2), a short version of the SF-36 covering eight subscales (physical functioning, role of physical health in limitations, bodily pain, general health, vitality, social functioning, role of emotional health in limitations and mental health). SF-12v2 scores range from 0 to 100, where higher scores represent a better quality of life. A mental component scale (MCS) and a physical component scale (PCS) are derived from the subscales by applying a normative scoring algorithm, where a score of 50 represents the norm of the general population, thus facilitating the interpretation of the results and the comparison across studies [29]. The SF-12v2 demonstrates adequate validity in patients with osteoarthritis and rheumatoid arthritis [30, 31]. Because the original orthogonal scoring algorithm described by Ware et al. [32] has received criticism due to inconsistencies with individual items of the instrument [33, 34], we used the oblique scoring algorithm suggested by Laucis et al. [33] with Canadian normative values identified by Hopman et al. [35]. The MCID in SF-12 for various populations of patients with musculoskeletal pain varies from 3.3 [36] to approximately 5 points [37].

### Analysis

Participant characteristics, waiting times and clinical outcomes were summarized using descriptive statistics (mean, standard deviation, median, interquartile range, frequency tables). Data were examined to identify missing values, outliers and coding errors. Patients’ baseline sociodemographic and clinical characteristics were compared between the three groups of waiting time (< 2 months, 2–6 months and >  6 months) using ANOVA for continuous variables and Chi-square tests for categorical variables. Similar analyses were conducted to compare patients with and without follow-up data at 6 months.

Associations between waiting time and change in clinical outcomes between the initial visit and the 6-month follow-up were tested using generalized estimating equations (GEE) [38]. GEE is an alternative approach to repeated measures ANOVA that is more flexible to missing values and non-normal distribution of outcomes [38]. This approach was chosen considering the expected high rates of patients with missing follow-up data [16]. A GEE with log link function was used to fit the skewed distribution of outcomes and an unstructured correlation matrix was specified. Post-hoc analyses were conducted to compare the change in outcomes between groups when a significant time X group interaction was found. Separate GEE models were computed for each dependent variable (change in BPI, NPRS, PCS or MCS). Sensitivity analyses were conducted to test de robustness of the models: additional GEE models were computed with a different categorization (five categories of waiting time) and multiple linear regression analyses were conducted with a continuous waiting time variable.

Patients’ sociodemographic and clinical characteristics known to be associated with the independent (waiting time) and dependent (BPI, NPRS, PCS, MCS) variables based on the literature and previous research by our team [13] were identified as potential confounding variables. Confounding variables were retained based on a combination of statistical approaches (i.e., backward and change-in-coefficient procedures), a literature review and the directed acyclic graph (DAG), an a priori theoretical approach [39], that were conducted independently (see Supplementary file). This combination of approaches allowed to benefit from their respective advantages and compensate their limitations [39–41]. Based on these procedures, the following variables were inserted in each adjusted GEE model: age, sex, pain duration, number of comorbidities and income. As a complement to GEE analyses, the proportion of patients reaching the MCID was compared across waiting time groups for each outcome using Chi-square tests.

Given that treatment options, potential for improvement, and waiting times may differ between rheumatic conditions, we conducted analyses to assess whether associations between waiting time and clinical outcomes differed for different rheumatic conditions. Separate GEE models were computed for patients who had one of two main diagnostic categories: osteoarthritis and fibromyalgia. Thus, analyses were successively computed for patients who only had osteoarthritis and then for patients who only had fibromyalgia in order to remove the effect of having other concomitant rheumatic conditions, as many patients had more than one. Analysis of patients with rheumatoid arthritis was not possible due to the small number of participants with this specific condition. Other categories (*Joint pain, effusion and other unspecified joint disorders only* and *Other rheumatic conditions or combinations of conditions*) were not retained for these secondary analyses because they included a variety of heterogenous diagnostic codes.

All statistical analyses were computed with IBM SPSS Statistics v.25.0 (IBM Corp., Armonk, NY, USA). The study was approved by the *Institut de réadaptation en déficience physique de Québec (IRDPQ)* Ethics Committee (#EMP-2015-449).

## Results

### Sample characteristics at baseline

**Fig. 1:**
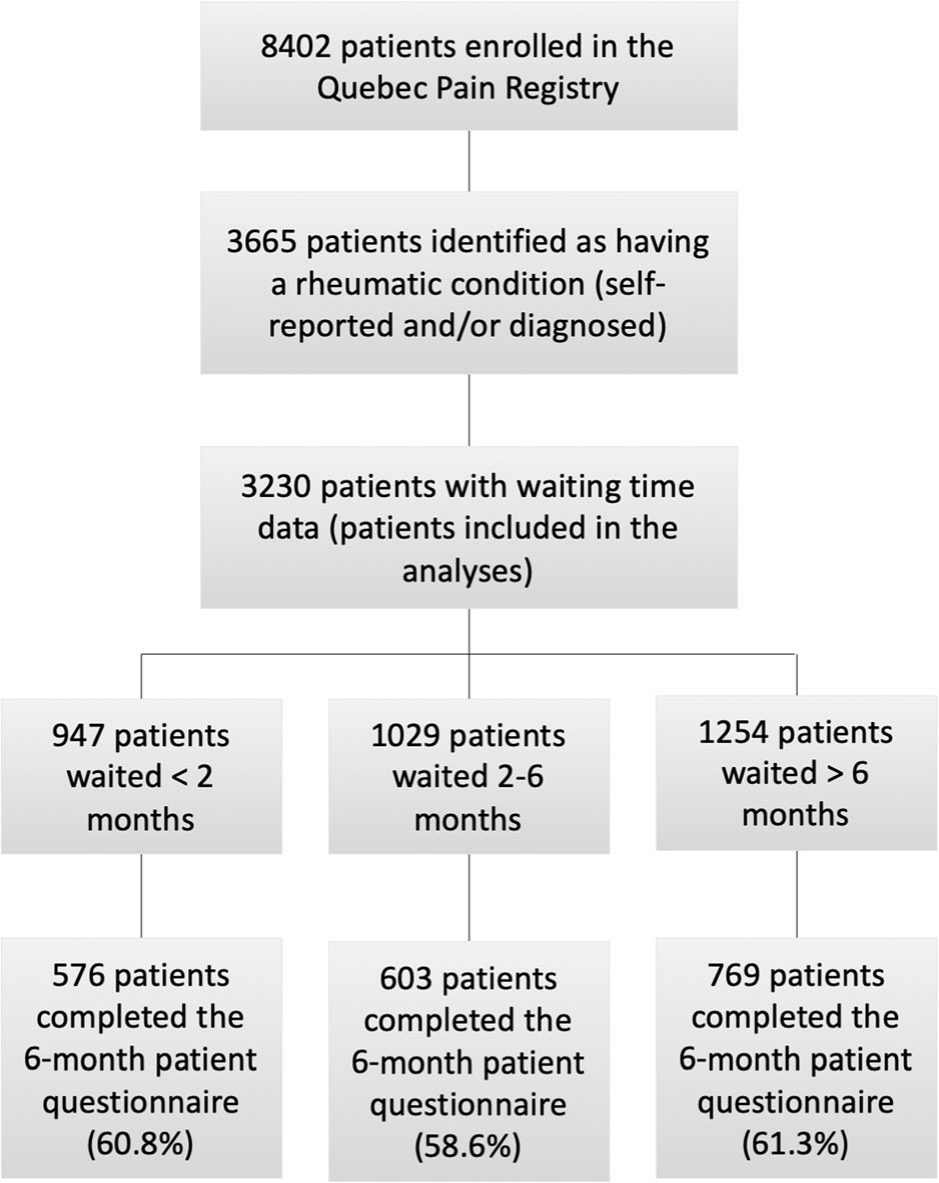
Patient flow diagram

Of the 8402 patients included in the Quebec Pain Registry, 3665 were identified as having a rheumatic condition. Of those 3665 patients, 3230 had valid waiting time data and were included in the analysis (see Fig. 1). Twenty-nine percent of patients waited < 2 months, 31.9% waited between 2 and 6 months and 38.8% waited > 6 months before their initial appointment at the MPTF.

Mean age of the patients was 55.8 ± 14.0 years (mean ± standard deviation) and the majority were women (66.2%) and Caucasian (92.6%). Thirty-six percent were on permanent or temporary disability and 45.6% had an annual household income of less than $35,000 CDN. Patients’ mean duration of pain was 6.8 ± 8.9 years (median = 3.2 years; interquartile range = 1.2–9.2). Self-reported depression (45.7%) and anxiety (41.7%) were highly prevalent. Twenty-one percent had osteoarthritis as their only rheumatic condition, 21.0% had only fibromyalgia and 1.3% had only rheumatoid arthritis (see Table 1). There were significant differences in age, sex, civil status, education, employment status, household income, pain duration, psychological comorbidities and diagnosis between waiting time groups (see Table 1).

**Table 1:** Baseline characteristics of patients with rheumatic conditions

	Full sample (*n* = 3230)	Waiting time groups			
		< 2 months (*n* = 947)	2–6 months (*n* = 1029)	> 6 months (*n* = 1254)	Group test *p* value ^d^
Age, mean (SD ^a^)	55.8 (14.0)	57.1 (15.0)	54.9 (13.9)	55.5 (13.4)	0.001
Sex, *n* (%)					0.032
Female	2138 (66.2)	641 (67.7)	648 (63.0)	849 (67.7)	
Male	1090 (33.7)	305 (32.2)	380 (36.9)	405 (32.3)	
Race/Ethnicity, *n* (%)					0.377
Caucasian	2991 (92.6)	882 (93,1)	943 (91,6)	1166 (93)	
Other	237 (7.3)	64 (6.8)	85 (8.3)	88 (7.0)	
Civil status, *n* (%)					0.004
Married or common law	1782 (55.2)	544 (57.4)	592 (57.5)	646 (51.5)	
Single, separated/divorced or widowed	1447 (44.8)	403 (42.6)	436 (42.4)	608 (48.5)	
Education, *n* (%)					0.005
Primary/None	321 (9.9)	96 (10.1)	89 (8.6)	136 (10.8)	
Secondary	1242 (38.5)	325 (34.3)	404 (39.3)	513 (40.9)	
CEGEP or Technical school	871 (27.0)	257 (27.1)	279 (27.1)	335 (26.7)	
University	791 (24.5)	266 (28.1)	255 (24.8)	270 (21.5)	
Employment status ^b^, *n* (%)					< 0.001
Full-time/part-time job	785 (24.3)	263 (27.8)	240 (23.3)	282 (22.5)	
On permanent disability	657 (20.3)	120 (12.7)	212 (20.6)	325 (25.9)	
On temporary disability	515 (15.9)	157 (16.6)	194 (18.9)	164 (13.1)	
Retired	787 (24.4)	270 (28.5)	223 (21.7)	294 (23.4)	
Other (including unemployed, student, homemaker and volunteer)	485 (15.0)	137 (14.5)	159 (15.5)	189 (15.1)	
Household income $CDN/year, *n* (%)					< 0.001
< 35,000	1474 (45.6)	368 (38.9)	448 (43.5)	658 (52.5)	
35,000-79,999	961 (29.8)	308 (32.5)	299 (29.1)	354 (28.2)	
≥ 80,000	416 (12.9)	147 (15.5)	149 (14.5)	120 (9.6)	
Pain duration (years), mean (SD)	6.8 (8.9)	5.0 (7.7)	6.8 (8.6)	8.3 (9.6)	< 0.001
Main psychological comorbidities					
Depression, *n* (%)	1477 (45.7)	369 (39.0)	474 (46.1)	634 (50.6)	< 0.001
Anxiety, *n* (%)	1348 (41.7)	355 (37.5)	422 (41.0)	571 (45.5)	0.001
Number of comorbidities, mean (SD)	3.0 (2.1)	2.8 (2.0)	3.0 (2.0)	3.2 (2.1)	< 0.001
Diagnosis, *n* (%)					< 0.001
Osteoarthritis only	668 (20.7)	199 (21.0)	214 (20.8)	255 (20.3)	
Fibromyalgia only	678 (21.0)	154 (16.3)	254 (24.7)	270 (21.5)	
Rheumatoid arthritis only	41 (1.3)	13 (1.4)	14 (1.4)	14 (1.1)	
Fibromyalgia and osteoarthritis	171 (5.3)	37 (3.9)	40 (3.9)	94 (7.5)	
Joint pain, effusion and other unspecified joint disorders only	693 (21.5)	260 (27.5)	198 (19.2)	235 (18.7)	
Other rheumatic conditions or combinations of conditions ^c^	979 (30.3)	284 (30.0)	309 (30.0)	386 (30.8)	

^a^ *SD* Standard deviation

^b^ This multiple-choice variable was recoded into a mutually exclusive variable. In cases of multiple answers, priority was given to the employed category and then on disability, retired and unemployed categories

^c^ Other NADW rheumatic conditions included: Spondylosis/spondylitis and allied disorders, Soft tissue disorders, Carpal tunnel syndrome, Diffuse connective tissue disease, Gout and other crystal arthropathies, Other specified rheumatic conditions

^d^ Group differences were tested using ANOVA for continuous variables and χ^2^ for categorical variables

Of the 3230 patients included in the analysis, 1948 (60.3%) responded to the 6-month follow-up patient questionnaire. The 1282 patients with missing follow-up data were slightly younger (54.6 vs. 56.5) and did not significantly differ from the rest of the sample at baseline in terms of sex, pain duration, BPI, SF-12v2 PCS and pain intensity scores.

### Association between waiting time and pain interference

An overall change of − 0.75 ± 2.05 (out of 10) in patients’ BPI scores was observed from baseline to 6 months (time effect *p* < 0.001). Only the < 2 months group reached a mean improvement above the MCID (1/10). Results from the GEE analyses (Table 2) showed waiting time was associated with change in BPI (adjusted time X group effect *p* < 0.001). In post-hoc analyses, the < 2 months group experienced a larger decrease in pain interference based on adjusted change in estimated marginal means (EMM) from baseline to 6 months than patients in the 2–6 months or the > 6 months groups (Table 2). However, the mean between-group differences in EMM were smaller than the MCID for these post-hoc analyses. There was no significant difference between the 2–6 months and > 6 months groups. A significantly higher proportion of patients in the < 2 months group reached the MCID (48.3%) compared to the 2–6 months (40.3%) and > 6 months groups (34.9%) (*p* < 0.001).

**Table 2:** Association between waiting time and the change in BPI from baseline to the 6-month follow-up, for the overall sample and by main rheumatic condition

	BPI at baseline, Mean (SD)	BPI at 6 months, Mean (SD)	GEE models			
			Unadj. ***p*** value ^**a**^	Unadj. change from baseline to 6 months, EMM (95% CI)	Adj. ***p*** value ^**a,b**^	Adj. change from baseline to 6 months, EMM ^**b**^ (95% CI)
**Overall sample (n)**	3228	1946	< 0.001		< 0.001	
Total	5.97 (2.13)	5.20 (2.48)				
< 2 months	5.77 (2.24)	4.61 (2.65)		−1.16 [− 1.45, − 0.87]		− 1.12 [− 1.42, − 0.81]
2–6 months	6.12 (2.10)	5.38 (2.39)		− 0.70 [− 0.91, − 0.48]		− 0.70 [− 0.93, − 0.48]
> 6 months	6.00 (2.07)	5.49 (2.34)		− 0.51 [− 0.69, − 0.32]		− 0.47 [− 0.66, − 0.28]
**Osteoarthritis (n)**	668	394				
Total	5.71 (2.19)	4.87 (2.59)	0.004		0.033	
< 2 months	5.73 (2.25)	4.33 (2.68)		− 1.40 [− 2.12, − 0.68]		− 1.24 [− 2.02, − 0.46]
2–6 months	5.82 (2.20)	5.05 (2.56)		−0.63 [− 1.15, − 0.11]		− 0.66 [− 1.19, − 0.12]
> 6 months	5.59 (2.13)	5.15 (2.50)		− 0.40 [− 0.85, 0.042]		−0.40 [− 0.85, 0.05]
**Fibromyalgia (n)**	677	359				
Total	6.13 (2.11)	5.52 (2.40)	0.003		0.002	
< 2 months	6.21 (2.05)	4.97 (2.44)		− 1.28 [− 1.98, −0.57]		− 1.30 [− 2.05, − 0.55]
2–6 months	6.02 (2.20)	5.31 (2.53)		−0.72 [− 1.20, − 0.24]		−0.76 [− 1.27, − 0.24]
> 6 months	6.20 (2.06)	6.02 (2.18)		− 0.35 [− 0.71, − 0.004]		−0.32 [− 0.65, 0.02]

*BPI* Brief pain inventory (average score on the 0–10 interference items), *SD* standard deviation, *GEE* generalized estimating equations, *EMM* estimated marginal means, *95% CI* 95% confidence interval

^a^ Time X group effect *p* value

^b^ Adjusted for age, sex, pain duration, number of comorbidities and income

### Association between waiting time and pain intensity

There was an overall change of − 0.8 ± 2.1 (out of 10) in pain intensity from baseline to 6 months (time effect *p* < 0.001) and there was a significant time X group effect (adjusted *p* < 0.001) (Table 3). Only the < 2 months group achieved an improvement above a MCID of 15%. Post-hoc analyses showed that the < 2 months group had a larger decrease in their pain intensity EMM than the 2–6 months or the > 6 months groups (Table 3). Again, the mean between-group differences in EMM were smaller than the MCID. There was no significant difference between the 2–6 months and > 6 months groups. A significantly greater proportion of patients in the < 2 months group (47.5%) achieved a decrease in pain intensity above the MCID compared to the 2–6 months (29.6%) and the > 6 months groups (28.4%) (*p* < 0.001).

**Table 3:** Association between waiting time and the change in pain intensity from baseline to the 6-month follow-up, for the overall sample and by main rheumatic condition

	Pain intensity at baseline, Mean (SD)	Pain intensity at 6 months, Mean (SD)	GEE models			
			Unadj. ***p*** value ^**a**^	Unadj. change from baseline to 6 months, EMM (95% CI)	Adj. ***p*** value ^**a,b**^	Adj. change from baseline to 6 months, EMM ^**b**^ (95% CI)
**Overall sample (n)**	3227	1948	< 0.001		< 0.001	
Total	7.0 (1.9)	6.2 (2.3)				
< 2 months	6.9 (2.0)	5.6 (2.5)		−1.3 [− 1.6, − 1.0]		−1.3 [− 1.6, − 0.9]
2–6 months	7.0 (1.8)	6.4 (2.2)		−0.6 [− 0.9, − 0.4]		−0.6 [− 0.9, − 0.4]
> 6 months	6.9 (1.9)	6.5 (2.1)		− 0.4 [− 0.6, − 0.3]		− 0.4 [− 0.6, − 0.2]
**Osteoarthritis (n)**	668	396	0.058		0.114	
Total	6.7 (1.9)	6.1 (2.4)				
< 2 months	6.6 (2.0)	5.6 (2.6)		−1.1 [− 1.8, − 0.4]		−1.1 [− 1.8, − 0.3]
2–6 months	6.9 (1.8)	6.3 (2.3)		−0.6 [− 1.1, 0.03]		−0.5 [− 1.1, 0.01]
> 6 months	6.6 (1.9)	6.2 (2.2)		− 0.4 [− 0.8, 0.05]		− 0.4 [− 0.9, 0.02]
**Fibromyalgia (n)**	676	359	0.087		0.022	
Total	6.9 (1.8)	6.5 (2.1)				
< 2 months	7.1 (1.6)	6.2 (2.3)		−0.9 [− 1.6, − 0.3]		− 1.0 [− 1.6, − 0.3]
2–6 months	6.9 (1.7)	6.4 (2.1)		− 0.5 [− 1.0, 0.02]		−0.5 [− 1.1, 0.01]
> 6 months	6.9 (1.9)	6.7 (2.0)		− 0.3 [− 0.7, 0.03]		−0.2 [− 0.6, 0.1]

*SD* standard deviation, *GEE* generalized estimating equations, *EMM* estimated marginal means, *95% CI* 95% confidence interval

^a^ Time X group effect *p* value

^**b**^ Adjusted for age, sex, pain duration, number of comorbidities and income

### Association between waiting time and health-related quality of life

An overall improvement over time was observed for the SF-12v2 PCS (2.3 ± 7.8) and MCS (2.2 ± 9.5) (time effects *p* < 0.001), but the mean improvement was clinically significant (MCID = 3.3 pts) only in the < 2 months group. GEE analyses showed a significant time X group effect (adjusted *p* ≤ 0.001) for both scales (Tables 4 and 5). Patients in the < 2 months group experienced a greater improvement (higher scores represent a better quality of life) in the two measures of health-related quality of life from baseline to 6 months compared to the groups with longer waiting time (Tables 4 and 5). The mean between-group differences in EMM were smaller than the MCID. There was no significant difference between patients who waited 2–6 months and those who waited > 6 months. A significantly larger proportion of patients in the < 2 months group (45.9%) reached the MCID for the PCS, compared to the 2–6 months group (37.6%) and in the > 6 months group (39.4%) (*p* = 0.010). For the MCS, no significant differences were observed, as 45.4% of patients in the < 2 months group reached the MCID, compared to 39.4% in the 2–6 months group and 40.2% in the > 6 months group (*p* = 0.078). Sensitivity analyses (GEE with five categories of waiting time and regression analyses) yielded similar results for all four outcomes (data not shown).

**Table 4:** Association between waiting time and the change in SF-12v2 physical component scale from baseline to the 6-month follow-up, for the overall sample and by main rheumatic condition

	PCS at baseline, Mean (SD)	PCS at 6 months, Mean (SD)	GEE models			
			Unadj. ***p*** value ^**a**^	Unadj. change from baseline to 6 months, EMM (95% CI)	Adj. ***p*** value ^**a,b**^	Adj. change from baseline to 6 months, EMM ^**b**^ (95% CI)
**Overall sample (n)**	3210	1940	< 0.001		< 0.001	
Total	23.5 (9.7)	26.1 (11.2)				
< 2 months	24.3 (10.1)	28.2 (12.3)		3.8 [2.7, 4.9]		3.9 [2.7, 5.1]
2–6 months	23.2 (9.8)	25.1 (10.6)		1.6 [0.7, 2.4]		1.6 [0.6, 2.5]
> 6 months	23.0 (9.4)	25.3 (10.6)		1.9 [1.2, 2.6]		2.0 [1.2, 2.8]
**Osteoarthritis (n)**	665	395	0.009		0.057	
Total	24.7 (9.7)	27.5 (11.2)				
< 2 months	24.1 (9.8)	28.4 (11.4)		4.7 [2.1, 7.3]		4.3 [1.6, 7.0]
2–6 months	24.6 (10.2)	27.0 (11.5)		1.7 [−0.4, 3.8]		1.8 [−0.6, 4.2]
> 6 months	25.3 (9.2)	27.2 (10.7)		1.7 [0.02, 3.34]		1.9 [0.1, 3.8]
**Fibromyalgia (n)**	670	358	0.049		0.038	
Total	22.7 (9.6)	25.0 (11.0)				
< 2 months	22.7 (8.8)	26.8 (11.7)		4.4 [1.6, 7.2]		4.7 [1.6, 7.8]
2–6 months	23.8 (10.1)	26.2 (11.3)		2.7 [0.8, 4.5]		2.3 [0.4, 4.3]
> 6 months	21.6 (9.5)	22.9 (9.9)		1.6 [0.1, 3.1]		1.5 [−0.1, 3.1]

*PCS* SF-12v2 Physical component scale, *SD* standard deviation, *GEE* generalized estimating equations, *EMM* estimated marginal means, *95% CI* 95% confidence interval

^a^ Time X group effect *p* value

^b^ Adjusted for age, sex, pain duration, number of comorbidities and income

**Table 5:** Association between waiting time and the change in SF-12v2 mental component scale from baseline to the 6-month follow-up, for the overall sample and by main rheumatic condition

	MCS at baseline, Mean (SD)	MCS at 6 months, Mean (SD)	GEE models			
			Unadj. ***p*** value ^**a**^	Unadj. change from baseline to 6 months, EMM (95% CI)	Adj. ***p*** value ^**a,b**^	Adj. change from baseline to 6 months, EMM ^**b**^ (95% CI)
**Overall sample (n)**	3210	1940	0.000		0.001	
Total	29.1 (11.4)	31.6 (12.5)				
< 2 months	29.9 (11.7)	33.5 (13.0)		3.6 [2.4, 4.9]		3.7 [2.3, 5.0]
2–6 months	28.7 (11.5)	30.5 (12.6)		1.4 [0.4, 2.4]		1.5 [0.3, 2.6]
> 6 months	28.8 (11.0)	31.0 (11.8)		1.9 [1.0, 2.8]		2.0 [1.0, 3.0]
**Osteoarthritis (n)**	665	395	0.112		0.308	
Total	31.4 (11.3)	33.5 (12.3)				
< 2 months	30.7 (11.4)	33.7 (12.1)		3.4 [0.7, 6.2]		3.2 [0.3, 6.2]
2–6 months	31.5 (11.9)	33.4 (13.3)		1.1 [−1.2, 3.4]		1.6 [−1.3, 4.4]
> 6 months	32.0 (10.6)	33.5 (11.5)		1.2 [−0.9, 3.29]		1.5 [−0.8, 3.7]
**Fibromyalgia (n)**	670	358	0.021		0.012	
Total	27.2 (11.0)	29.0 (12.6)				
< 2 months	27.9 (10.5)	32.7 (12.2)		4.9 [1.7, 8.1]		4.9 [1.5, 8.3]
2–6 months	27.7 (11.8)	28.9 (13.7)		1.6 [−0.6, 3.8]		0.9 [−1.1, 3.0]
> 6 months	26.3 (10.5)	27.1 (11.4)		1.3 [−0.6, 3.21]		1.0 [−1.0, 2.9]

*MCS* SF-12v2 Mental component scale, *SD* standard deviation, *GEE* generalized estimating equations, *EMM* estimated marginal means, *95% CI* 95% confidence interval

^a^ Time X group effect *p* value

^b^ Adjusted for age, sex, pain duration, number of comorbidities and income

### Association between waiting time and patient outcomes for specific rheumatic conditions

The association between waiting time and the outcome measures in patients with only osteoarthritis and patients with only fibromyalgia are presented in Tables 2, 3, 4 and 5. In patients with osteoarthritis, there was a significant difference observed in the change in BPI (adjusted time X group effect *p* < 0.05) between waiting time groups, with the < 2 months group having a larger improvement in pain interference compared to groups with longer waiting time. Again, the differences in improvement between groups were smaller than the MCID and there was no significant difference in change in BPI between patients who waited 2–6 months and those who waited > 6 months. There were no significant time X group effects in changes in pain intensity and SF-12v2 mental component scale for the targeted rheumatic condition. Analyses for the SF-12v2 physical component scale yielded opposite results, with significant unadjusted time X group effect and non-significant adjusted time X group effect.

Analyses with fibromyalgia patients found statistically significant between-group differences below the MCID in mean changes (EMM) in BPI, SF-12v2 physical component scale and SF-12v2 mental component scale (adjusted time X group effect *p* < 0.05). The < 2 months group experienced a greater improvement in these outcomes from baseline to 6 months compared to the groups with longer waiting time, while no difference was found between the last two groups. For pain intensity, unadjusted and adjusted models showed non-significant and significant effects, respectively.

## Discussion

Chronic pain is a major issue in many patients with rheumatic diseases and its management often requires timely multidisciplinary pain treatment. In this study, more than two third of patients waited over 2 months before their initial visit to the MPTF, including 39% who waited more than 6 months. This is consistent with the findings of another Canadian study, in which 35% of patients waited more than 6 months before their initial MPTF visit [16]. There were significant differences in patients’ characteristics between waiting time groups, including household income. This reflects the result of another article on factors associated with waiting time, which raised ethical issues regarding the inequity of access to services based on income [13].

The present study examined the association between waiting time for multidisciplinary pain treatment and clinical outcomes in patients with rheumatic conditions. The results revealed statistically significant differences in improvement in clinical outcomes between waiting time groups, with patients waiting less than 2 months averaging a larger improvement in all clinical outcomes compared to patients who waited 2–6 months or over 6 months before their initial visit. This larger improvement for patients waiting less than 2 months was, however, of small magnitude and may not have had a clinically important impact on all patients. Although the differences in improvement between groups were smaller than the MCID for all outcomes, there was a significantly higher proportion of patients achieving improvements above the MCID in BPI, pain intensity, SF-12v2 PCS in the group of patients waiting less than 2 months compared to the other groups.

Few other studies examined the impact of waiting time for health care services on the clinical progression of patients with musculoskeletal conditions. In two studies conducted with patients with lumbar stenosis waiting for spinal surgery, a longer waiting time was significantly associated with a smaller improvement in pain, disability, quality of life and mental health after surgery [42, 43]. Lynch et al.’s systematic review found that patients waiting longer than 6 months for treatment for various chronic pain conditions experienced a deterioration while waiting in terms of health-related quality of life and psychological health of patients [14].

These findings suggest the need for potential strategies to reduce waiting times and to mitigate the impact of waiting times on patients’ condition, including the implementation of preclinic group education sessions [44] and self-management interventions while waiting [45]. Prioritization processes in MPTF could also be investigated and improved by developing best practice guidelines (e.g., recommending a structured referral template) [46]. Resources and recommendations could be made available to patients with lower priority and their referring physicians [47].

The present study also highlights the small magnitude of improvement experienced by patients in terms of pain interference, pain intensity and health-related quality of life from the initial visit to the 6-month follow-up, with only patients waiting less than 2 months averaging clinically significant improvements. Such marginal clinical gains were also reported by Pagé et al. in their study on pain intensity trajectories and predictors of outcomes in MPTF [48]. In that study, patients with various chronic pain conditions reported a small decrease in pain intensity (NPRS) from 6.95 ± 1.7 at baseline to 6.21 ± 2.2 at 6 months as well as a minimal improvement in pain interference (BPI), from 5.98 ± 2.0 at baseline to 5.29 ± 2.4 at 6 months [48]. The authors found that only one quarter of patients with chronic pain experienced a significant improvement in pain intensity [48]. In our study, the limited improvement experienced by patients after receiving care in MPTF may be explained by the chronic and non-curative nature of most rheumatic conditions, and the fact that patients experienced pain for an average 7 years before receiving services in MPTF. Still, the limited effectiveness of treatments is a concern for patients, clinicians and pain clinic administrators, and should be taken into consideration when re-examining chronic pain services organization. Although this reflection is beyond the scope of this article, we advise that actions are taken not only to improve access to services, but also their effectiveness. For example, innovative long-term pain management programs building on collaboration with the primary care teams could be tested and implemented as an alternative to punctual specialized interventions.

In light of the small improvement found in this study, one may argue that the long duration of pain experienced by patients makes a few additional months of waiting insignificant. Nevertheless, we hypothesize that in most cases, their condition progressively deteriorated to the point where a timely specialized multidisciplinary pain management approach was considered necessary by their referring physician. The high level of pain intensity and interference and the low quality of life reported in the present study suggest that patients suffered a significant burden while waiting for MPTF and thus may be affected by a delay of several months.

The small magnitude of improvement in treatment outcomes in our study suggests the need to re-evaluate current pain management approaches for persons with rheumatic conditions in MPTF as well as earlier in the care trajectory (e.g., primary care). Upstream interventions could be developed and implemented. For example, education and self-management resources could be offered by primary care providers to improve management of the disease in its early stage. The use of online support groups, pain management videos, blogs [49] or eConsult services [50], as well as facilitating communication between family physicians and pain specialists, could improve pain treatment for patients with rheumatic conditions. In addition, the large number of patients referred to MPTF for osteoarthritis or fibromyalgia conditions suggests that primary care failed to effectively manage these patients. To our knowledge, few primary care settings in Canada offer coordinated interprofessional chronic pain treatment that include psychotherapy, physiotherapy and self-management interventions.

Our secondary objective was to determine if the association between waiting time and clinical outcomes was different for specific rheumatic conditions. Approximately 21% of the sample had osteoarthritis as their only rheumatic condition, and the same proportion had fibromyalgia only. In patients with osteoarthritis, the results showed a statistically significant but not clinically important group difference in terms of improvement in pain interference, with patients waiting less than 2 months having a greater improvement compared to patients with longer waiting time. There were no between-group differences in terms of pain intensity and health-related quality of life for patients with osteoarthritis. Analyses conducted with patients having fibromyalgia revealed statistically significant but not clinically important group differences in changes in pain interference and health-related quality of life from baseline to 6 months, again favoring patients who waited less than 2 months. There were no differences between waiting time groups regarding pain intensity. These findings may have implications for patients with fibromyalgia, who often face longer waiting times before receiving services in MPTF compared to patients with other rheumatic conditions [13].

### Limitations

A first limitation of this study was the high proportion of patients lost to follow-up. Despite the fact that patients with missing follow-up data did not differ from the rest of the sample in most sociodemographic and clinical characteristics, there was a risk of selection bias. Consequently, the choice of using GEE analyses provided a suitable approach with respect to missing follow-up data [38]. In addition, the data were collected between 2008 and 2014 and it is possible that changes to MPTF organization may have occurred after that period. However, based on feedback received from experts and clinicians in the field, there is no indication that waiting time have been significantly improved in Quebec’s MPTF. Other limitations inherent to most patient registry studies included potential inaccuracy of self-reported data, coding errors or inconsistency in the data collection procedures. Also, the selection of patients with rheumatic conditions from the Quebec Pain Registry was not limited to the primary diagnosis as the Registry data did not allow to distinguish a primary diagnosis from secondary diagnoses. Thus, it is possible that we included patients with rheumatic conditions whose primary diagnosis was a non-rheumatic condition. With the interpretation of the findings, it must thus be taken into account that a rheumatic comorbidity, not being the primary diagnosis, may affect the patient’s overall pain experience in a different way. Furthermore, because our study focussed on patients with rheumatic conditions, further studies will need to investigate the impact of waiting time for patients with other musculoskeletal diagnosis seen in MPTF, such as non-rheumatic low back pain.

Another possible limitation was the categorization of the waiting time variable in three groups instead of using a continuous variable. This may have affected the strength of the associations found with the clinical outcomes. However, GEE analyses conducted with a different categorization (five categories of waiting time) and regression analyses with a continuous waiting time variable yielded similar results and did not change the conclusions. In addition, our study did not take into account the waiting time prior to the MPTF referral (e.g., delays before the diagnosis, before the physician appointment, before reception of the referral). Patients may face multiple delays along their care trajectories that may affect the progression of their condition. Also, patients with rheumatic conditions may experience fluctuations in their symptoms during the wait. Such fluctuations could have influenced the results, since they were likely to be referred to the pain clinic at a point when they reported worst symptoms. The data available did not allow us from drawing conclusions regarding the evolution of symptoms *during* the waiting time (no data collection at the time of referral). However, considering patients’ high pain intensity and interference and their low quality of life at baseline, it is less likely that a significant decrease of symptoms occurred before the initial visit. Also, mean baseline outcome measures were similar between waiting time groups, which may be an indication that symptom intensity was stable regardless of the time since referral.

Lastly, prioritization processes used in MPTF, which were not documented in the Quebec Pain Registry, may have influenced the results. For example, some MPTF may have considered the potential for improvement as a prioritization criterion. Hence, patients identified with a limited potential for improvement may have received a lower priority level, leading to a longer waiting time. We believe this limitation was nonetheless attenuated by controlling for pain duration, age and comorbidities, three characteristics that likely influence the potential for improvement.

## Conclusion

In conclusion, patients face long delays before receiving services in MPTF. Longer delays were significantly associated with less improvement in pain interference, pain intensity and health-related quality of life. However, differences were below the minimal clinically important difference. In addition, there was a significantly higher proportion of patients achieving clinically significant improvements in the group of patients waiting less than 2 months compared to patients waiting longer. The effect of waiting time on patients’ condition deserves the attention of clinicians, MPTF administrators and policy-makers. Our study suggests possible variations between types of rheumatic conditions, with more consistent effects seen in patients with fibromyalgia than in patients with osteoarthritis. Future studies are needed to investigate the impact of waiting time on patients’ condition prior to the admission to the pain clinic, that is from the point of referral to the initial visit. Finally, the findings suggest the need to investigate and implement strategies to reduce waiting times and improve outcomes, for example self-management interventions, prioritization of referrals and increased resource allocation.

## Supplementary information

**Additional file 1.** Method to identify confounding variables.

## Data Availability

The data supporting the findings of this study are available from the Quebec Pain Registry, propriety of the Quebec Pain Research Network, a network supported by the Quebec Health Research Funds (FRQ-S). Restrictions apply to the availability of these data, which were used under license for the current study, and so are not publicly available. Data could be made available from the authors upon a formal request made to the Quebec Pain Registry management team. This request will have to follow the policy rules of the Quebec Pain Registry, therefore the request must be supported by a peer-reviewed research protocol and will need to be approved by the Quebec Pain Registry scientific committee and the *Centre hospitalier de l’Université de Montréal* (CHUM) research center Institutional Review Board.

## Abbreviations

BPI: Brief Pain Inventory
CI: Confidence interval
DAG: Directed acyclic graph
EMM: Estimated marginal means
ICD-9-CM: International Classification of Diseases, Ninth revision, Clinical Modification
GEE: Generalized estimating equations
MCID: Minimal clinically important difference
MCS: Mental component scale
MPTF: Multidisciplinary pain treatment facility
NADW: National Arthritis Data Workgroup
NPRS: Numeric pain rating scale
PCS: Physical component scale
QPR: Quebec Pain Registry
SD: Standard deviation
SF-12v2: 12-item Short-Form Health Survey version 2
