# Supplementary material for: The association between waiting time and multidisciplinary pain treatment outcomes in patients with rheumatic conditions
Source: BMC Rheumatol. 2020 Oct 23;4:59. doi: 10.1186/s41927-020-00157-0 (PMC7583241; doi:10.1186/s41927-020-00157-0)
Supplement: Supplementary file 1 — Additional file 1. Method to identify confounding variables. [file 41927_2020_157_MOESM1_ESM.docx]

**Supplementary file 1: Method to identify confounding variables**

The following approaches were used independently to select relevant confounding variables among these characteristics. This method allowed to benefit from multiple approaches’ advantages and compensate their limitations.

| **Approach** | **Description** | **Variables retained** |
| --- | --- | --- |
| Theoretical approach | Variables identified based on a literature review on factors associated with pain outcomes or waiting time. | Age  Sex  Pain duration  Household income*  Comorbidities  Education* |
| Bivariate analyses  (see Table 1) | Variables identified based on their association with waiting time and the clinical outcome using bivariate analyses (ANOVA and linear regression) | Age  Household income*  Comorbidities  Education* |
| Change-in-coefficient approach  (see Table 2) | Variables identified based on their association with waiting time and clinical outcomes using multiple linear regression analyses. Variables resulting in a 10% change of the R^2^ coefficient were retained. | Age  Household income*  Comorbidities  Education* |
| *Directed acyclic graph* (DAG)  (see Figure) | This approach allows to select a combination of confounding variables that minimize bias using an *a priori* theoretical process. | Age  Pain duration  Household income |

*The variables “education” and “household income” were considered redundant (risk for multicollinearity). The latter was retained because it was found to be significantly associated with waiting time in another study by our team.

**Table 1. Results from the bivariate analyses***

| **Variable** | **p-value** |
| --- | --- |
| Age | < 0.001 |
| Sex | 0,498 |
| Household income | < 0.001 |
| Comorbidities | < 0.001 |
| Education | < 0.001 |

*Based on ANOVA analyses for categorical variables and linear regressions for continuous variables, with the Brief pain inventory (BPI) at baseline as the outcome of interest

**Table 2. Results from the change-in-coefficient approach***

| **Included** | **Excluded** |
| --- | --- |
| Age | Sex |
| Household income | Pain duration |
| Comorbidities |  |
| Education |  |

*Variables were selected based on a multiple linear regression analysis with backward selection if they induced a 10% change in the R^2^ coefficient, with the Brief pain inventory (BPI) at baseline as the outcome of interest and waiting time a predictor

**
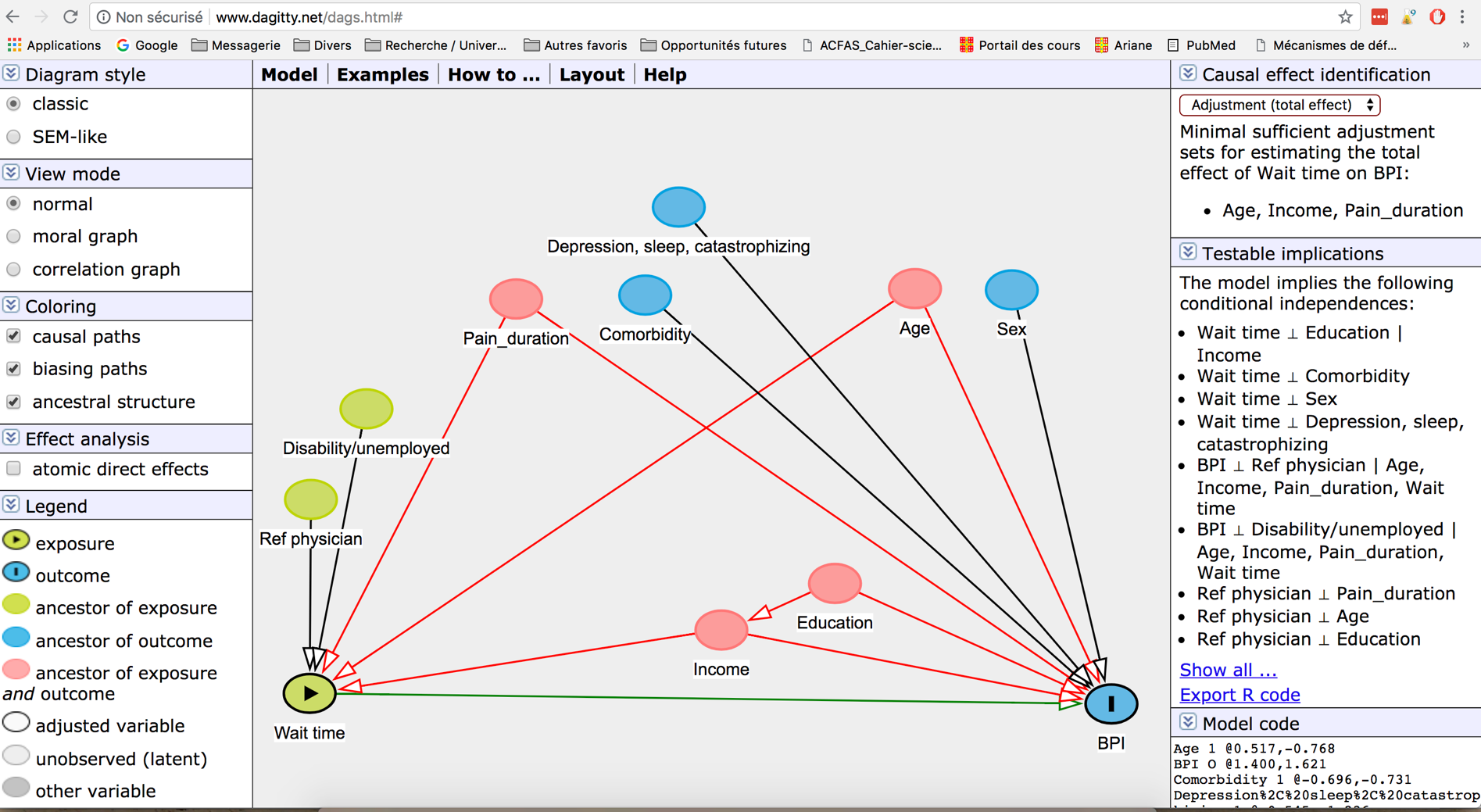
**

**Figure**. Results of the *Directed acyclic graph* (DAG) process. [www.dagitty.net](http://www.dagitty.net/)


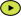
 exposure


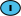
 outcome


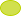
 ancestor of exposure


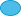
 ancestor of outcome


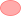
 ancestor of exposure *and* outcome
